# The Effect of a Future-Self Avatar Mobile Health Intervention (FutureMe) on Physical Activity and Food Purchases: Randomized Controlled Trial

**DOI:** 10.2196/32487

**Published:** 2022-07-07

**Authors:** Annette Mönninghoff, Klaus Fuchs, Jing Wu, Jan Albert, Simon Mayer

**Affiliations:** 1 Institute for Mobility University of St. Gallen St Gallen Switzerland; 2 Institute for Customer Insight University of St. Gallen St Gallen Switzerland; 3 ETH AI Center ETH Zurich Zurich Switzerland; 4 Institute for Computer Science University of St. Gallen St Gallen Switzerland

**Keywords:** mHealth, mobile health, preventative medicine, avatar, present bias, nutrition tracking, physical activity, randomized controlled trial

## Abstract

**Background:**

Insufficient physical activity and unhealthy diets are contributing to the rise in noncommunicable diseases. Preventative mobile health (mHealth) interventions may help reverse this trend, but present bias might reduce their effectiveness. Future-self avatar interventions have resulted in behavior change in related fields, yet evidence of whether such interventions can change health behavior is lacking.

**Objective:**

We aimed to investigate the impact of a future-self avatar mHealth intervention on physical activity and food purchasing behavior and examine the feasibility of a novel automated nutrition tracking system. We also aimed to understand how this intervention impacts related attitudinal and motivational constructs.

**Methods:**

We conducted a 12-week parallel randomized controlled trial (RCT), followed by semistructured interviews. German-speaking smartphone users aged ≥18 years living in Switzerland and using at least one of the two leading Swiss grocery loyalty cards, were recruited for the trial. Data were collected from November 2020 to April 2021. The intervention group received the FutureMe intervention, a physical activity and food purchase tracking mobile phone app that uses a future-self avatar as the primary interface and provides participants with personalized food basket analysis and shopping tips. The control group received a conventional text- and graphic-based primary interface intervention. We pioneered a novel system to track nutrition by leveraging digital receipts from loyalty card data and analyzing food purchases in a fully automated way. Data were consolidated in 4-week intervals, and nonparametric tests were conducted to test for within- and between-group differences.

**Results:**

We recruited 167 participants, and 95 eligible participants were randomized into either the intervention (n=42) or control group (n=53). The median age was 44 years (IQR 19), and the gender ratio was balanced (female 52/95, 55%). Attrition was unexpectedly high with only 30 participants completing the intervention, negatively impacting the statistical power. The FutureMe intervention led to small statistically insignificant increases in physical activity (median +242 steps/day) and small insignificant improvements in the nutritional quality of food purchases (median −1.28 British Food Standards Agency Nutrient Profiling System Dietary Index points) at the end of the intervention. Intrinsic motivation significantly increased (*P*=.03) in the FutureMe group, but decreased in the control group. Outcome expectancy directionally increased in the FutureMe group, but decreased in the control group. Leveraging loyalty card data to track the nutritional quality of food purchases was found to be a feasible and accepted fully automated nutrition tracking system.

**Conclusions:**

Preventative future-self avatar mHealth interventions promise to encourage improvements in physical activity and food purchasing behavior in healthy population groups. A full-powered RCT is needed to confirm this preliminary evidence and to investigate how future-self avatars might be modified to reduce attrition, overcome present bias, and promote sustainable behavior change.

**Trial Registration:**

ClinicalTrials.gov NCT04505124; https://clinicaltrials.gov/ct2/show/NCT04505124

## Introduction

### Noncommunicable Diseases and Risk Factors

Noncommunicable diseases (NCDs) are today’s leading causes of death. They are responsible for over half of the years lived in disability and drive 59%-80% of total health care costs in affluent developed countries like New Zealand and Switzerland [1-3]. Among others, insufficient physical activity and an unhealthy sodium-rich diet are recognized as key risk factors for NCDs by the World Health Organization (WHO) [4]. Increased salt consumption has been shown to increase blood pressure, while sodium-rich, low-fruit, and low–whole-grain diets may increase the risk of death from cardiovascular diseases, cancer, and diabetes [5-7]. Insufficient physical activity has been linked to increased risks of cancer, cardiovascular diseases, diabetes, dementia, and depression [8-11]. To combat the rise in NCDs, the WHO has released a global action plan providing clear recommendations on the required physical activity levels and maximum salt consumption [4]. However, the latest studies showed that globally, 28% of adults do not meet the WHO’s physical activity guidelines [12]. Moreover, salt intake was approximately twice the recommended amount in 2010 [13], and has not improved significantly since [14].

### Physical Activity and Salt Reduction Mobile Health Interventions

Wide-scale prevention programs are needed to make significant improvements toward realizing the WHO’s NCD action plan. Mobile health (mHealth) interventions can make an important contribution toward efficiently reaching the wider population. While numerous physical activity mHealth interventions have proven effective in a wide range of populations [15-18], their effectiveness in decreasing salt consumption is less clear [19]. Preliminary results suggest that while mHealth interventions might lower participants’ salt consumption by raising awareness, the quality of the evidence gathered in such studies is very low. Scholars have also criticized the lack of rigorous research designs [19]. In addition, mHealth nutrition interventions, such as those aiming to reduce sodium intake, regularly experience low user adoption and high attrition [20-22]. A review of commercially available nutrition-tracking mHealth apps showed that most such interventions relied on manual or semiautomated tracking technologies [23], and required significant time investment from users, favoring underreporting [24] and high attrition rates as a result. There is a lack of studies employing improved technological means, such as automated digital receipt processing [25,26], image analysis, and sensor-based tracking. Thus, there is a need for high-quality research that evaluates automated tracking systems in rigorous research settings [23,27].

### Challenges of Preventative mHealth Programs

Preventative mHealth interventions (ie, mHealth interventions targeting healthy population groups) have been shown to be less effective than curative mHealth interventions (ie, mHealth interventions targeting sick or at-risk population groups) [15,18]. The reasons behind this moderating effect are manifold and remain little explored. The Health Action Process Approach (HAPA) is a widely used and validated theoretical framework that can serve as a starting point for explaining differences in intervention effectiveness [28]. According to the associated model, health behavior change is best described as a 2-phase process consisting of a preintentional motivational phase and a postintentional volitional phase [28]. Within the first phase, risk awareness (ie, the awareness that one’s behavior may cause a health risk), positive outcome expectancies (ie, the belief that a change in behavior will lead to reduced health risk), and action self-efficacy (ie, the belief in one’s ability to perform preventative health behavior) are fundamental for intention formation [28]. However, present bias may cause low risk awareness and decrease positive outcome expectancies in healthy population groups and thus limit the effectiveness of preventative mHealth interventions [29]. Present bias describes a situation where people take suboptimal decisions as they over-discount future gains [29-32]. When facing preventative health decisions, consumers are asked to make unpleasant behavior changes today (ie, eating more vegetables instead of meat and exercising instead of watching movies), while the benefits of their behaviors occur in the future (ie, better future health and longer life expectancy). In reverse, the negative consequences of a risky lifestyle are not immediately evident but only occur with a time lag, hindering the formation of positive outcome expectancies (ie, low symptom salience) [33].

### Future-Self Avatars to Overcome Present Bias and Increase Risk Awareness

Existing research argues that immediate monetary incentives can help overcome present bias in health decision-making and improve adaptation of preventative mHealth interventions [34-36]. However, extrinsic monetary incentives might lead to motivational crowding-out and diminish intrinsic motivation for behavior change over time [37,38]. Alternative strategies to overcome present bias and to increase risk awareness have been developed in related fields, such as pension planning [31] and taxation [39]. A little explored possible solution is the use of future-self avatars (ie, avatars that confront users with an aged version of themselves). Such avatars have been shown to increase users’ tendency to accept later gains over immediate ones [31]. Evidence on the impact of avatars on health decision-making is still scarce, but preliminary results indicate that avatars can be leveraged to increase physical activity in obese populations [40], to promote smoking cessation in young adults [41], to reduce sodium intake [42], and to help manage depression [43]. To date, however, no study has investigated the impact of a preventative future-self avatar mHealth intervention in a randomized controlled trial (RCT) setting.

### Objectives

Accordingly, the primary aim of this study was 2 fold. First, the study aimed to understand the impact of a preventative future-self avatar mHealth intervention on physical activity and on the nutritional quality of food purchases. Second, it aimed to test the acceptability of a fully automated nutrition tracking system leveraging grocery receipts in an RCT. Our secondary aim was to understand the impact of a future-self avatar preventative mHealth intervention on motivational and recovery self-efficacy, outcome expectancy, and types of motivation.

## Methods

### Study Design

We conducted our study in cooperation with a large Swiss health insurance company that facilitated participant recruitment. A 12-week parallel RCT design was employed, followed by semistructured interviews with a selection of 15 participants who completed the trial. Recruitment began in November 2020 and ended in December 2020. Data were collected from November 2020 to April 2021. Potential participants were contacted via the insurance company’s email newsletter and informed about the study, eligibility criteria, and data privacy policies. Additionally, social media networks were leveraged to recruit trial participants. Potential participants received a direct link to the iOS or Android app store where they could download the app-based intervention. After downloading the mobile app, answering a survey on eligibility criteria, and consenting to the data privacy statement, participants were randomized into an intervention or a control group (1:1 ratio) via a random allocation algorithm programmed into the app. Participants did not receive any information about the existence of 2 different app versions (intervention and control). Apart from the in-depth interviews, data collection was fully automated, thus preventing reporting, detection, and performance bias. The semistructured interviews were conducted by trained researchers who were neither the primary researchers nor the authors of this study. Selection of interview participants was blinded, meaning that interviewers had no knowledge of the interviewees’ group allocation or the outcome data collected in the RCT. The trial has been registered on ClinicalTrials.gov (NCT04505124), and the study has been reported in accordance with the CONSORT guidelines [44].

### Study Population

Eligible participants were smartphone users aged ≥18 years, living in Switzerland, and German-speaking. Participants had to be participating in at least one of the two leading grocery loyalty card programs in Switzerland (Multimedia Appendix 1). They were asked to confirm that they had no medical condition that prevented increased levels of physical activity or changes in daily nutrition.

### FutureMe Intervention

**Figure 1:**
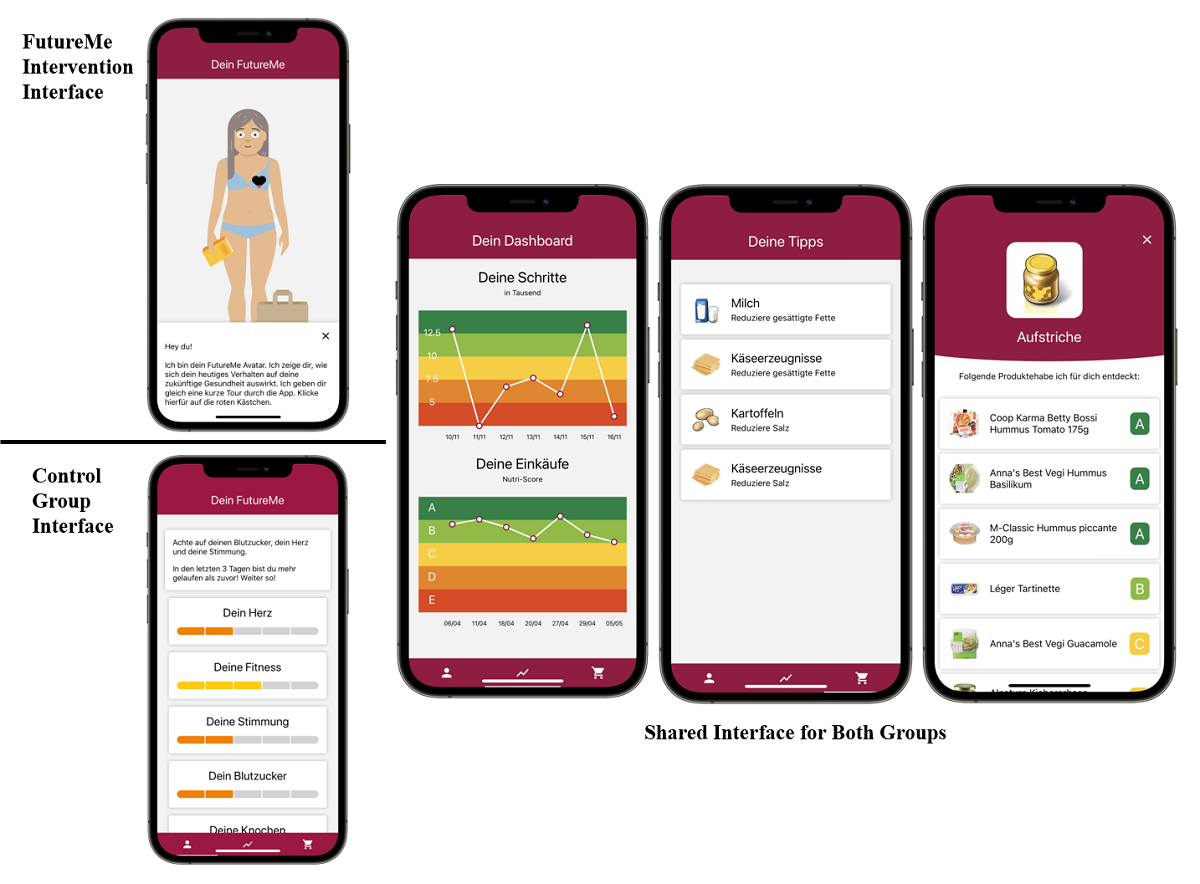
Overview of the FutureMe and control intervention mobile apps.

The FutureMe app aims to promote the overall nutritional quality of participants’ food purchases and to encourage increases in physical activity to ≥7500 steps per day, based on prior findings that health benefits can be achieved at this level [45-47]. Participants allocated to the FutureMe intervention received the FutureMe app. This comprised a future-self avatar, a dashboard displaying the number of daily steps and the nutritional quality of food purchases, a personalized basket analysis showing food categories with the highest improvement potentials, and personalized product recommendations of healthier product alternatives within the food categories purchased by the participant (Figure 1). All app components were updated daily, ensuring that participants received the most recent data. The intervention was fully automated and required no human-to-human interaction, making it highly scalable, tailored, and cost-effective [15].

The personalized basket analysis and product recommendations were based on the British Food Standards Agency Nutrient Profiling System Dietary Index (FSA-NPS DI), which is also referred to as Nutri-Score [48]. The intervention mechanism was co-designed with the Swiss Society for Nutrition and aimed to induce a behavior shift toward healthier choices within the frequently purchased categories that contributed most negatively to a user’s recent FSA-NPS DI assessment. Concretely, in the FutureMe intervention, we evaluated the 7 most recently purchased baskets using FSA-NPS DI points and assessed the contribution of negative points (ie, sugar, sodium, saturated fats, and energy density) by each food category. Specifically, we calculated the weight-averaged contribution of each food category across the 4 negative FSA-NPS DI dimensions. We identified up to four food categories (out of the 125 food categories used in the food composition database of the study) that contributed most negatively to a user’s FSA-NPS DI score. The FutureMe app then recommended healthier product alternatives within these problematic categories. The presented product recommendations were meant to help users make healthier choices by substituting frequently consumed and rather unhealthy food items with alternatives of higher nutritional quality, without requiring users to modify the structure of their diets or key dietary habits (eg, recommending lower-salt cheese alternatives). In addition to showing healthier product alternatives, users received relevant nutritional tips that were adapted based on the identified problematic food categories.

Although no scholarly consensus toward a single metric to capture the nutritional quality of food purchases exists [48], the Nutri-Score framework and the underlying established FSA-NPS DI system allow for a reliable heuristic to assess the improvement potentials of purchased grocery baskets, thus enabling automated personalized purchase recommendations for a specific user.

We selected the Nutri-Score framework as a monitoring proxy for the nutritional quality of recently purchased baskets and as an intervention mechanism to support healthier food choices within the FutureMe app for multiple reasons. First, the FSA-NPS DI has been identified as a useful and validated tool to discriminate individuals according to the quality of the diet, allowing the monitoring of dietary change [49]. Second, within a comparative study among purchase quality indicators in Switzerland, the Nutri-Score framework has been validated to estimate diet activities from shopping data better than other purchase indicators [50]. Third, when compared to other front-of-package labels, the Nutri-Score label has been validated to show superior effectiveness among consumers, especially among the at-risk population [51]. Fourth, although the Nutri-Score framework was developed for rating individual products and not aggregated shopping baskets, its scoring mechanism has been proven to correlate with overall dietary behavior when applied on grocery purchase data sets [50].

To the best of our knowledge, although the FSA-NPS DI and Nutri-Score frameworks are established concepts, applying them within a smartphone-mediated intervention design using digital receipts from loyalty card data has not been done before.

### Future-Self Avatar

**Figure 2:**
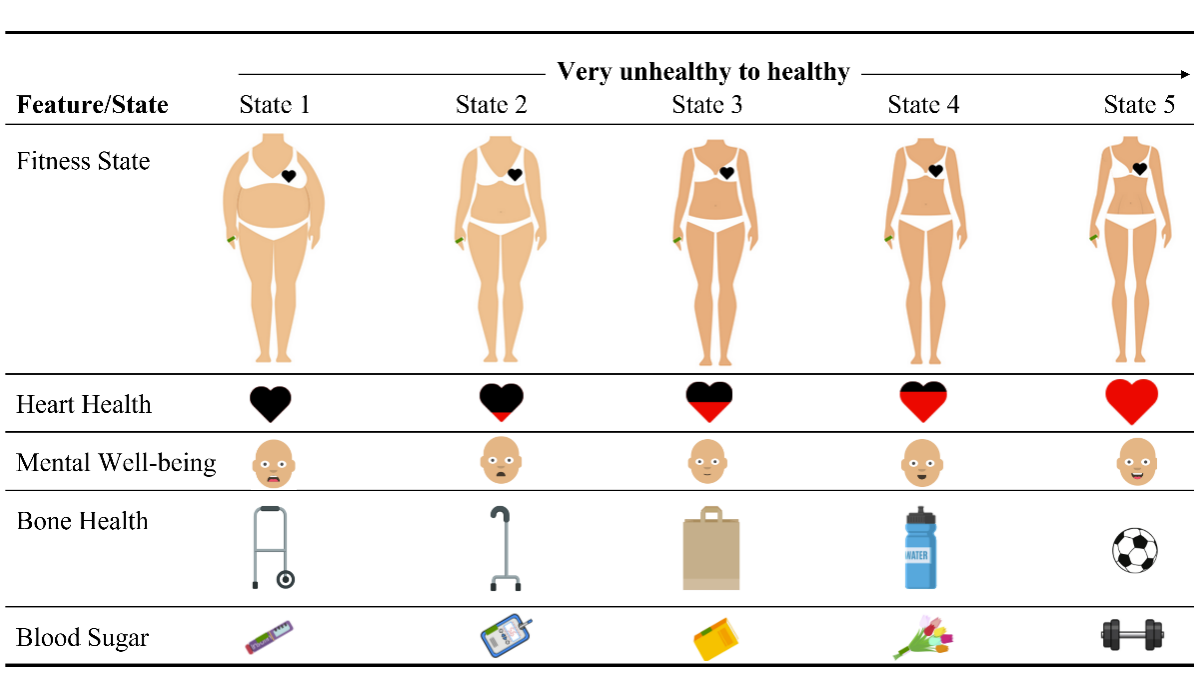
Avatar feature states.

The future-self avatar was designed based on the HAPA behavior change model and encouraging findings of a prior pilot study by Fuchs et al [42]. The future-self avatar aims to increase participants’ awareness of the future health consequences of their current nutritional and activity behaviors in a fun and engaging way (cf, risk perception, outcome expectancy, and self-efficacy [28]). It thus seeks to minimize the negative impact of present bias [31]. Participants could personalize their avatar during the sign-up process to ensure that the avatar best resembled themselves, in order to increase personal relevance and to positively impact health behavior change [52]. The avatar was depicted using a cartoon-style unanimated 2D design to avoid feelings of eeriness and repulsion as described by the uncanny valley effect [53]. When first opening the app, participants were exposed to an aging simulation in which their avatar aged +20 years compared to their current age. The future-self avatar had 5 features that consumers could affect with their food purchases and physical activity behavior, with each feature having 5 different states (see Table 1 and Figure 2). As the nutritional recommendations of our research were based on the FSA score framework [53], the avatar health features captured 6 nutritional subcategories of this framework. The health features were based on scientific literature but were also designed to be easily understood by the broad group of trial participants. The calculation scheme for each state is provided in Multimedia Appendix 2.

**Table 1:** System rules defining the feature states of the future-self avatar.

Feature and behavioral influencer		Weighting	Calculation scheme	Unit
**Fitness state**				
	Physical activity	100%	Average past 7 days	Steps/day
**Heart health**				
	Sodium	50%	Average past 12 baskets	FSA-NPS DI^a^ points
	Saturated fatty acids	50%	Average past 12 baskets	FSA-NPS DI points
**Mental well-being**				
	Fruits and vegetables	50%	Average past 12 baskets	FSA-NPS DI points
	Fiber	50%	Average past 12 baskets	FSA-NPS DI points
**Bone health**				
	Protein	100%	Average past 12 baskets	FSA-NPS DI points
**Blood sugar**				
	Sugar	100%	Average past 12 baskets	FSA-NPS DI points

^a^FSA-NPS DI: British Food Standards Agency Nutrient Profiling System Dietary Index [53].

**Figure 3:**
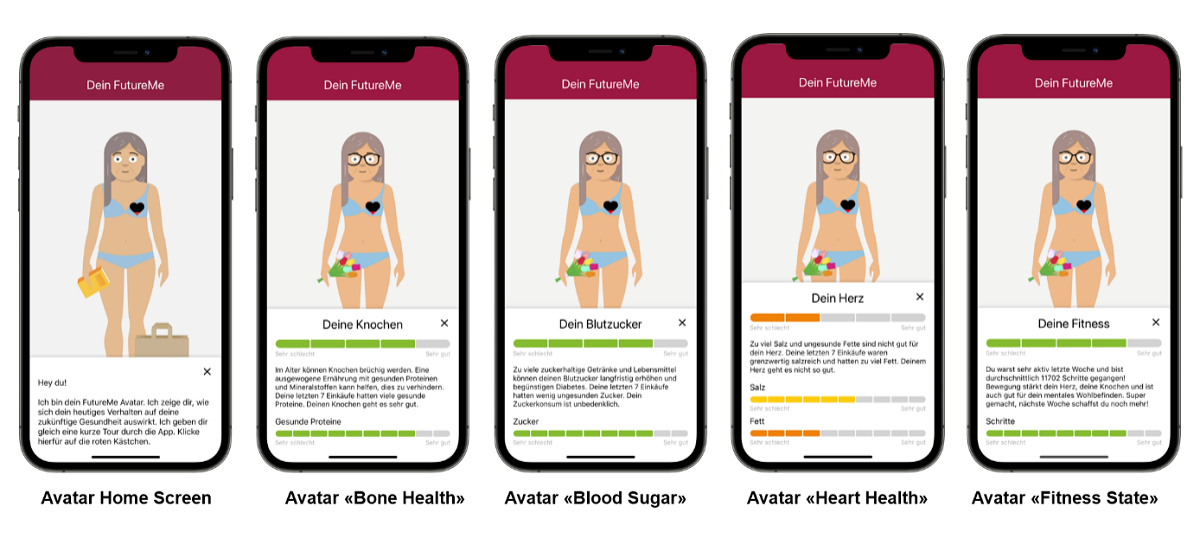
Overview of avatar features.

The 5 features were clickable. If clicked, a pop-up screen opened, providing users with consequential health feedback and how their individual past behavior had impacted the feature. Participants were provided with an encouraging message to improve the respective health behavior (eg, “You were very active last week and took an average 11,702 steps/day! Physical activity improves your heart health, strengthens your bones, and also benefits your mental well-being! Well done, next week you can do even more” for *Fitness State*; Figure 3).

### Fully Automated Nutrition and Physical Activity Tracking

Nutrition and physical activity tracking within the FutureMe intervention app pioneered a novel and fully automated tracking system. Food purchases recorded via loyalty cards were used as a proxy for nutrition, as South African studies leveraging credit card data have shown this to be a promising method for changing everyday health behavior [25,26]. We gathered food purchase data using the loyalty cards from Switzerland’s 2 leading grocery retailers. Data were analyzed using a comprehensive product database comprising over 55,000 products and their nutritional information. Participants were requested to connect their loyalty cards once while signing up. Step data were collected by the accelerometer on participants’ mobile phones.

### Control Intervention

Apart from the future-self avatar, the control intervention was identical to the FutureMe intervention (see Figure 1). Instead of the future-self avatar, the control app used a more traditional text- and graphics-based interface to provide consequential health feedback to participants (see Figure 1). The control intervention used the same automated data tracking system as the FutureMe intervention.

### Outcomes and Measurement

Demographic information as well as attitudinal and motivational constructs were collected at baseline (T0), and after completing the 12-week intervention (T3), using an online survey that was programmed into the mHealth app. Step and food purchase data were collected at baseline (T0) and continuously throughout the 12-week intervention period using the fully automated tracking system previously described. At baseline, we collected steps per day for the past 6 days prior to sign-up and data for all grocery baskets of the past 2 years that were registered on the loyalty cards. User engagement was recorded continuously for 12 weeks after completing the sign-up process. Continuously collected data were aggregated into three 4-week periods (T1, T2, and T3). Qualitative data were collected from a group of randomly selected participants in semistructured video interviews after the 12-week intervention period (T3).

The primary outcomes were as follows:

1. Physical activity (steps/day) was measured objectively to avoid reporting biases [54]. Step information was collected through the built-in accelerometer in iOS or Android mobile phones, as these devices are widely owned and require no additional investment [55], and thus offer a scalable solution to track physical activity. Mobile phones have been shown to reliably measure steps if carried continuously by participants and when steps are taken at a modest to fast pace [55-57].
2. The nutritional quality of food purchases was measured based on the British Food Standards Agency Nutrient Profiling System [49,50] (in FSA-NPS DI points; –15 most healthy to +40 least healthy) [58]. Nutritional quality was calculated by separating solid foods and beverages, in accordance with the calculation method suggested by Julia et al [59]. We only report results for solid foods.

The secondary outcomes were the nutritional subcategories comprising the Nutri-Score framework (secondary outcomes 1-6) [60], user engagement (secondary outcome 7), and attitudinal and motivational constructs (secondary outcomes 8-12) as follows:

1. Sugars, excluding fructose and lactose, in grams (g) per 100 g food purchases (in FSA-NPS DI points; 0 most healthy to +10 least healthy).
2. Saturated fatty acids in g per 100 g food purchases (in FSA-NPS DI points; 0 most healthy to +10 least healthy).
3. Sodium in mg per 100 g food purchases (in FSA-NPS DI points; 0 most healthy to +10 least healthy).
4. Fruits, vegetables, legumes, and nuts in % per 100 g food purchases (in FSA-NPS DI points; 0 least healthy to +5 most healthy).
5. Fiber in g per 100 g food purchases (in FSA-NPS DI points; 0 least healthy to +5 most healthy).
6. Protein in g per 100 g food purchases (in FSA-NPS DI points; 0 least healthy to +5 most healthy).
7. User engagement (app logins/week).
8. Motivational self-efficacy, which was measured with an adjusted 4-item scale adopted from Schwarzer et al [61] on a 7-point Likert scale (1 [completely disagree] to 7 [completely agree]). The scale measured participants’ confidence in their capability to be physically active and shop healthily in general (eg, “In my everyday life, I know how to shop healthily”) (Multimedia Appendix 3). With regard to internal consistency, Cronbach α was .728.
9. Recovery self-efficacy, which was measured with an adjusted 2-item scale adopted from Schwarzer et al [61] on a 7-point Likert scale (1 [completely disagree] to 7 [completely agree]). The scale measured participants’ confidence in their capability to be physically active and shop healthily after a setback (eg, “After my vacation, I’m sure that I’ll go back to balanced shopping, even if I have to get used to it again”) (Multimedia Appendix 3). With regard to internal consistency, Cronbach α was .652.
10. Outcome expectancy, which was measured with an adjusted 6-item outcome expectancy scale adopted from Renner and Schwarzer [62] on a 7-point Likert scale (1 [completely disagree] to 7 [completely agree]) that measured participants’ expectations about how their present behavior will impact their future health (eg, “I believe that I can positively influence my health in old age with my current exercise and shopping behavior”) (Multimedia Appendix 3). With regard to internal consistency, Cronbach α was .934.
11. Intrinsic motivation, which was measured with an adjusted 3-item autonomous motivation scale [63] on a 7-point Likert scale (1 [completely disagree] to 7 [completely agree]). The scale measured the degree to which participants were intrinsically motivated to be physically active and shop healthily (eg, “I exercise and shop healthily because I personally believe it’s best for my health”) (Multimedia Appendix 3). With regard to internal consistency, Cronbach α was .838.
12. Extrinsic motivation, which was measured with an adjusted 3-item controlled motivation scale [63] on a 7-point Likert scale (1 [completely disagree] to 7 [completely agree]). The scale measured the degree to which participants were extrinsically motivated to be physically active and shop healthily (eg, “I exercise and shop healthily because I want to see positive metrics on my activity tracker and health app”) (Multimedia Appendix 3). With regard to internal consistency, Cronbach α was .690.

In addition to the quantitative outcomes, we collected qualitative data in 15 semistructured video interviews. Data were gathered on app usefulness and ease of use, as these factors have been shown to impact technology acceptance [64]. Furthermore, data were collected on the relationship that participants formed with the avatar and on areas for improving the FutureMe intervention in order to guide future research.

### Sample Size

No other studies have so far compared a future-self avatar interface with a more conventional self-monitoring interface regarding the ability to motivate both physical activity increases and nutrition improvements in a healthy population sample. Given the limited availability of mHealth studies reporting Nutri-Score improvements based on shopping data, we focused on results from comparable mHealth physical activity studies to calculate the sample size. A systematic review of mHealth physical activity interventions found the step increase at the end of the intervention in a mixed population sample at 1566 steps [15] compared with both active and passive control groups. Baseline activity measurements for healthy population samples have been reported to range from 6745 to 6994 (SD 2422 to 2620) steps/day [65,66]. Using an α of .05 with a power of 80%, we estimated the minimum sample size for our trial to be 74-88 participants [67]. Dropout rates in mHealth studies vary significantly, depending on intervention duration and intervention components. A comparable 12-week mHealth physical activity study using scalable intervention components without human-to-human interaction reported a dropout rate of 22.4% [68]. Anticipating a 20% dropout rate, we inflated the sample size to 100.

### Statistical Analysis

Nonparametric tests were conducted to examine intervention effects. A Wilcoxon signed-rank test was used to compare the outcome variables before and after the intervention to test for within-group effects. To test for between-group differences between the FutureMe and control groups, we conducted Mann-Whitney *U* tests or Pearson chi-square tests. The level of significance was .05. Analyses were performed with SPSS Statistics 27 (IBM Corp) and Python 3.8.5 (Python Software Foundation).

Continuously measured data (physical activity, nutritional quality of food purchases, nutritional subcategories, and user engagement) were analyzed in 4-week periods, with T1 representing the mean value summarizing weeks 1-4, T2 summarizing weeks 5-8, and T3 summarizing weeks 9-12. Baseline values (T0) were also summarized depending on the outcome variable and data availability. For physical activity, T0 represents the mean steps per day of the 6 days prior to trial commencement. For the nutritional quality of food purchases and the nutritional subcategories, T0 represents all foods purchased within 4 weeks prior to trial commencement.

Sensitivity analysis was conducted for demographic variables and primary outcomes to test for randomness of missing data and attrition bias [69,70]. Missing data were not replaced given the limitations of imputation methods in samples with high attrition where data are missing at random [69]. To test for intervention effects and statistical differences, we used complete case analysis.

### Qualitative Data Analysis

All 15 semistructured video interviews were audio recorded, fully transcribed, coded, and analyzed following the principles of thematic content analysis [71]. Additionally, demographic information on all 15 interview candidates was quantitatively summarized.

### Ethics Approval

Participants gave their electronic informed consent to participate in the study prior to commencement. The Ethics Committee of the University of St. Gallen approved the protocol, recruitment strategies, and data privacy policies of this study (HSG-EC-2020-06-12-A).

## Results

### Demographic Data and Baseline Characteristics

**Figure 4:**
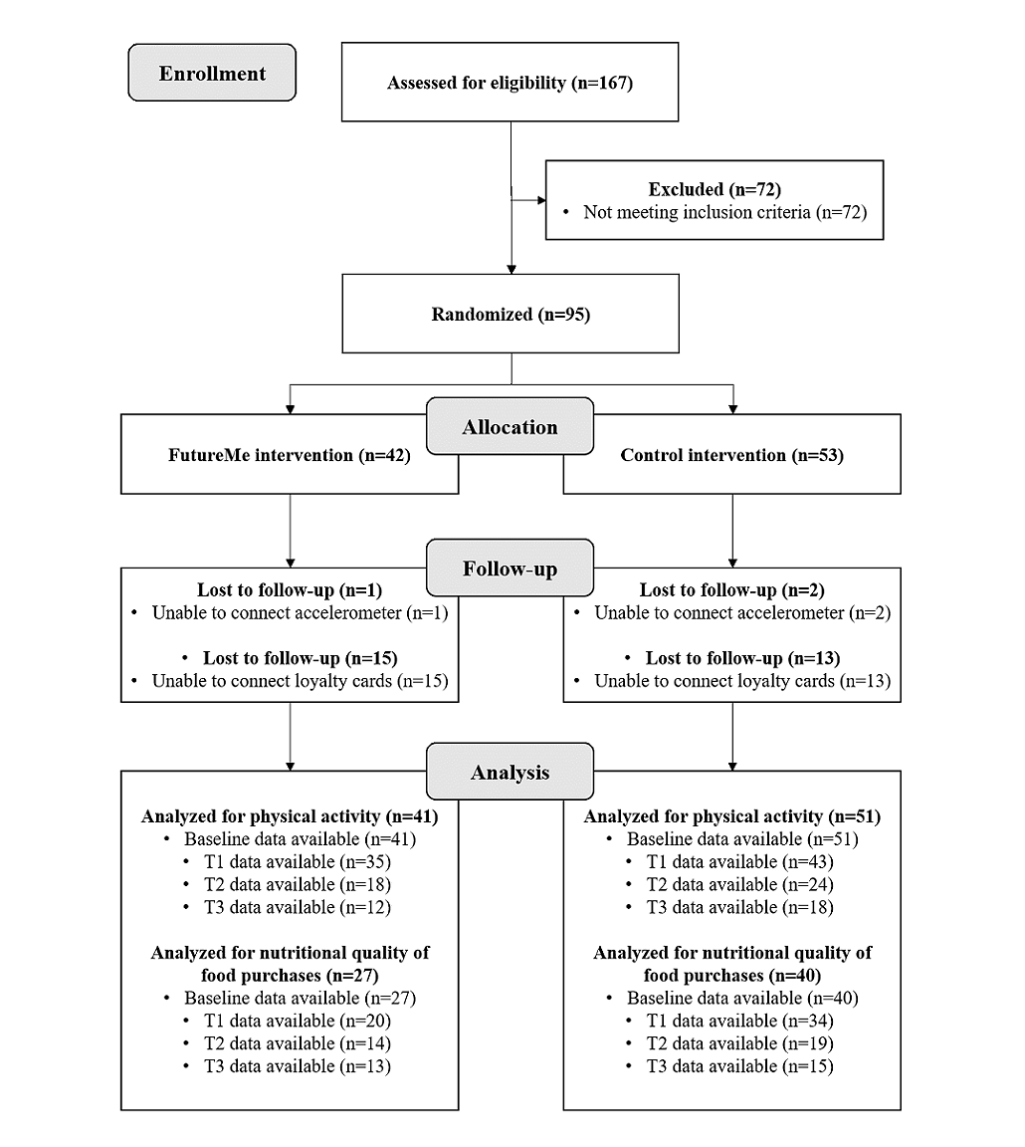
CONSORT flow chart. Limited data were available at T1-3 as some participants from both groups stopped using the FutureMe/control app. Data were only automatically pulled when users logged into the app.

As shown in the CONSORT flow chart (Figure 4), 167 participants downloaded the FutureMe/Control app from November 11 to December 15, 2020. Seventy-two participants were excluded as they did not meet the inclusion criteria. Ninety-five participants were randomized to either the FutureMe intervention (n=42) or the control intervention (n=53).

One participant in the intervention group and 2 participants in the control group were lost as they were unable to connect their mobile phone accelerometer to the intervention/control app. Fifteen participants in the intervention group and 13 participants in the control group were lost as they were unable to connect their loyalty cards to the FutureMe app. We saw unexpectedly high attrition during the 12-week intervention period (n=95, 68.4%), with only 13 participants from the intervention group and 18 participants from the control group using the app during the last 4 weeks of the trial.

As shown in Table 2, the median age of the participants was 44 years (IQR 19). The gender ratio was balanced, with 55% (52/95) female participants. The median household size was 2 (IQR 2), and most participants had normal body weight (56/95, 59%). The majority (76/95, 80%) of participants were recruited into the trial through the online panel of a Swiss health insurance company. No baseline differences existed between the intervention and control groups with regard to demographic data.

**Table 2:** Demographic data at baseline.

Characteristic		Total population (N=95)	Intervention group (n=42)	Control group (n=53)	*P* value
**Gender, n (%)**					>.99^a^
	Male	43 (45)	19 (45)	24 (45)	
	Female	52 (55)	23 (55)	29 (55)	
Age (years), median (IQR)		44 (19)	47 (15)	42 (25)	.46^b^
**BMI (kg/m^2^), n (%)**					.96^a^
	>30	11 (12)	5 (12)	6 (11)	
	25-30	28 (29)	12 (29)	16 (30)	
	<25	56 (59)	25 (60)	31 (58)	
Household size, median (IQR)		2 (2)	2 (2)	3 (2)	.61^b^
**Recruiting source, n (%)**					.50^a^
	Helsana	76 (80)	33 (79)	43 (81)	
	University	5 (5)	1 (2)	4 (8)	
	Social media	6 (6)	3 (7)	3 (6)	
	Other	8 (8)	5 (12)	3 (6)	

^a^Pearson chi-square test.

^b^Mann-Whitney *U* test.

At baseline, the median steps per day were 4624 (IQR 4497.79), that is, roughly half the number of steps per day compared with a representative Swiss sample [72]. The median nutritional quality of food purchases was 6.13 FSA-NPS DI points (IQR 4.02), which is in line with the values reported in a large-scale study [73]. Food purchases were low sugar (median 0.88 FSA-NPS DI points, IQR 1.20) and contained moderate amounts of saturated fatty acids (median 3.01 FSA-NPS DI points, IQR 1.66), sodium (median 2.50 FSA-NPS DI points, IQR 1.40), and protein (median 3.16 FSA-NPS DI points, IQR 0.93). The shares of fruits, vegetables, legumes, and nuts, and fiber were rather low (median 0.30 FSA-NPS DI points, IQR 0.48 and median 1.53 FSA-NPS DI points, IQR 0.77, respectively). No baseline differences were found between the intervention and control groups with regard to physical activity or the total nutritional quality of food purchases. Participants in the FutureMe intervention had significantly higher amounts of saturated fatty acids in their food purchases (*P*=.03) compared with the findings in the control group. Overall, participants showed high levels of motivational and recovery self-efficacy (median 6.0, IQR 1.25 and median 6.0, IQR 1.50, respectively), as well as outcome expectancy (median 6.2, IQR 1.60). Participants in the intervention group scored significantly higher on recovery self-efficacy than those in the control group (*P*=.03). Participants exhibited high intrinsic motivation to live a healthy lifestyle (median 6.0, IQR 1.33) and were less extrinsically motivated (median 3.0, IQR 2.33). Baseline characteristics with regard to all outcomes are presented in Table 3.

**Table 3:** Primary and secondary outcomes at baseline.

Primary and secondary outcomes			Total population				Intervention group					Control group					*P* value^a^
			Value, median (IQR)		n		Value, median (IQR)		n		Value, median (IQR)			n			
**Physical activity**																	
	Walking (steps/day)	4624.00 (4497.79)		92		4314.83 (3620.33)		41		5084.83 (5220.33)			51		.53		
**Nutritional quality of food purchases**																	
	Total nutritional quality (FSA-NPS DI^b^ points)^c^	6.13 (4.02)		67		6.73 (5.03)		27		5.83 (4.11)			40		.14		
**Nutritional subcategories**																	
	Sugars (FSA-NPS DI points)^d^	0.88 (1.20)		67		0.83 (1.08)		27		0.91 (1.31)			40		.73		
	Saturated fatty acids (FSA-NPS DI points)^d^	3.01 (1.66)		67		3.82 (2.83)		27		2.93 (1.37)			40		.03		
	Sodium (FSA-NPS DI points)^d^	2.50 (1.40)		67		2.77 (1.56)		27		2.42 (1.37)			40		.42		
	Fruits, vegetables, legumes, and nuts (FSA-NPS DI points)^e^	0.30 (0.48)		67		0.28 (0.53)		27		0.31 (0.47)			40		.64		
	Fiber (FSA-NPS DI points)^e^	1.53 (0.77)		67		1.49 (0.70)		27		1.56 (0.83)			40		.85		
	Protein (FSA-NPS DI points)^e^	3.16 (0.93)		67		3.20 (0.70)		27		3.15(1.03)			40		.31		
**Attitudinal and motivational constructs**																	
	Motivational self-efficacy^f^	6.00 (1.25)		95		6.00 (1.31)		42		6.00 (1.50)			53		.65		
	Recovery self-efficacy^f^	6.00 (1.50)		95		6.00 (1.50)		42		6.00 (1.50)			53		.03		
	Outcome expectancy^f^	6.20 (1.60)		95		6.10 (1.65)		42		6.20 (1.60)			53		.31		
	Intrinsic motivation^f^	6.00 (1.33)		95		5.83 (1.75)		42		6.00 (1.50)			53		.13		
	Extrinsic motivation^f^	3.00 (2.33)		95		2.67 (2.00)		42		3.33 (2.00)			53		.65		

^a^Mann-Whitney *U* test.

^b^FSA-NPS DI: British Food Standards Agency Nutrient Profiling System Dietary Index.

^c^FSA-NPS DI point scale: −15 most healthy to +40 least healthy.

^d^FSA-NPS DI point scale: 0 most healthy to +10 least healthy.

^e^FSA-NPS DI point scale: 0 least healthy to +5 most healthy.

^f^Likert scale: 1 [completely disagree] to 7 [completely agree].

### Physical Activity

As shown in Table 4, participants from both groups increased their physical activity levels within the first 4 weeks of the intervention (FutureMe T1: median 4577.85 steps/day, IQR 4620.26; control T1: median 5361.50 steps/day, IQR 5960.88). Participants in the intervention group consistently increased their physical activity over the course of the intervention from median 4314.81 (IQR 3620.33) steps/day at baseline to median 6042.31 (IQR 6242.31) steps/day at T2 and to median 4556.94 (IQR 5226.94) steps/day at the end of the intervention. In contrast, in the control group, the number of steps per day decreased after a short-term increase in T1, from median 5084.83 (IQR 5220.33) steps/day at baseline to median 2909.32 (IQR 5128.09) steps/day at T2 and to median 4821.64 (IQR 5228.79) steps/day at the end of the intervention. However, for both groups, the changes in the number of steps over the course of the intervention were not statistically significant. There were no significant differences in steps per day evident between the intervention and control groups.

**Table 4:** Primary outcomes by timepoint and group.

Primary outcome variables and groups		Baseline (T0)^a^		T1^b^			T2^c^			T3^d^	
		Value, median (IQR)	n	Value, median (IQR)	n	Value, median (IQR)		n	Value, median (IQR)		n
**Physical activity, walking (steps/day)**											
	Avatar	4314.83 (3620.33)	41	4577.85 (4620.26)	35	6042.45 (6242.31)		18	4556.94 (5226.94)		12
	Control	5084.83 (5220.33)	51	5361.50 (5960.88)	43	4909.32 (5128.09)		24	4821.64 (5228.79)		18
**Nutritional quality of food purchases, total nutritional quality (FSA-NPS DI^e^points)^f^**											
	Avatar	6.73 (5.03)	27	6.79 (3.67)	20	6.14 (2.63)		14	5.45 (3.19)		13
	Control	5.83 (4.11)	40	5.79 (5.41)	34	4.42 (5.42)		19	4.86 (3.75)		14

^a^Baseline: For steps, baseline is defined as average steps per day 6 days prior to enrolling in the trial, and for shopping-related outcome variables, baseline is defined as the nutritional value of all foods purchased within the 4 weeks before the trial.

^b^T1: week 1-4 average values.

^c^T2: week 5-8 average values.

^d^T3: week 9-12 average values (end of study).

^e^FSA-NPS DI: British Food Standards Agency Nutrient Profiling System Dietary Index.

^f^FSA-NPS DI point scale: −15 most healthy to +40 least healthy.

### Nutritional Quality of Food Purchases

Participants in both groups improved the nutritional quality of their food purchases over the course of the intervention (Multimedia Appendix 4), albeit insignificantly. Participants in the intervention group shopped less healthily within the first 4 weeks of the intervention (median 6.79 FSA-NPS DI points, IQR 3.67) compared with the findings at baseline (median 6.73 FSA-NPS DI points, IQR 5.03). However, they improved their shopping behavior in T2 (median 6.14 FSA-NPS DI points, IQR 2.63) and at the end of the intervention (median 5.45 FSA-NPS DI points, IQR 3.19). Participants in the control group consistently improved their shopping behavior over the course of the intervention from median 5.83 FSA-NPS DI points (IQR 4.11) at baseline to median 5.70 FSA-NPS DI points (IQR 5.31) at T1, median 4.42 FSA-NPS DI points (IQR 5.42) at T3, and median 4.86 FSA-NPS DI points (IQR 3.75) at the end of the intervention. At the end of the intervention, the control group purchased significantly healthier food than the FutureMe group (*P*=.02). However, the control group’s food baskets were already healthier at baseline, although not at statistically significant levels.

### Nutritional Subcategories

Multimedia Appendix 5 provides a summary of all secondary outcomes for all relevant timepoints. It also presents the results of the Wilcoxon signed-rank tests and Mann-Whitney *U* tests. Across both groups, no statistically significant improvements were found in nutritional subcategories during the intervention. As for directional developments, for the control group, improvements in the nutritional quality of food purchases were driven by reductions in sugar per 100 g food purchases between baseline (median 0.91 FSA-NPS DI points, IQR 1.31) and the end of the study (median 0.55 FSA-NPS DI points, IQR 1.07). For the intervention group, improvements in the nutritional quality of food purchases were driven by reductions in saturated fatty acids and sodium between baseline (saturated fatty acids: median 3.82 FSA-NPS DI points, IQR 2.83; sodium: median 2.77 FSA-NPS DI points, IQR 1.56) and the end of the study (saturated fatty acids: median 3.82 FSA-NPS DI points, IQR 2.83; sodium: median 2.77 FSA-NPS DI points, IQR 1.56). In both groups, the amounts of protein, fruits, vegetables, and fiber per 100 g in food purchases were relatively stable throughout the trial or even decreased slightly. At the end of the intervention, the control group purchased significantly healthier food than the FutureMe group with regard to sugar (*P*=.01) and saturated fatty acids (*P*=.01). However, the control group’s food baskets already contained significantly lower amounts of saturated fatty acids at baseline (*P*=.03).

### User Engagement

User engagement was similar across the FutureMe and control interventions (see Multimedia Appendix 5). Engagement was the highest during the first 4 weeks of the intervention (T1), where the FutureMe and control groups recorded a median of 5 logins per week (IQR 10) and 4 logins per week (IQR 10), respectively. User engagement decreased to 1 login per week (IQR 3) for the FutureMe group for T2 and remained flat (IQR 1) until the end of the study. For the control group, user engagement also dropped to a median of 1 login per week (IQR 3) at T2 but increased to 2 logins per week (IQR 2) at the end of the study.

### Attitudinal and Motivational Constructs

Multimedia Appendix 5 provides a summary of all attitudinal and motivational constructs preintervention and postintervention. It also presents the results of the Wilcoxon signed-rank tests and the Mann-Whitney *U* tests. Intrinsic motivation to lead a healthy lifestyle significantly (*P*=.03) increased from a median value of 5.83 (IQR 1.75) at baseline to 6.0 (IQR 1.67) at the end of the study for participants in the FutureMe group. In contrast, intrinsic motivation decreased throughout the intervention in the control group. The extrinsic motivation to lead a healthy lifestyle increased in both groups from baseline (FutureMe: median 2.67, IQR 2.00; control: median 3.33, IQR 2.00) to the end of the study (FutureMe: median 3.67, IQR 2.50; control: median 4.00, IQR 2.08). However, the changes were not statistically significant. At the end of the study, the FutureMe group showed significantly (*P*=.03) higher levels of recovery self-efficacy (median 7.00, IQR 1.00) compared with the levels in the control group (median 5.67, IQR 1.25). Motivational self-efficacy did not change significantly in either group. Outcome expectancy increased in the FutureMe group between baseline (median 6.10, IQR 1.65) and the end of the study (median 7.00, IQR 2.08). However, the change was not statistically significant. In contrast, outcome expectancy decreased in the control group between baseline (median 6.20, IQR 1.60) and the end of the study (median 5.67, IQR 1.62), although not at statistically significant levels.

### Sensitivity Analysis

To test for attrition bias and randomness of missing data, we compared demographic and primary outcome variables at baseline (T0) between participants who did not finish the trial (ie, dropouts) and participants who completed the trial (ie, trial completers). The results are presented in Table 5. We found no statistical differences between dropouts and trial completers with respect to our primary outcome variables, indicating a low risk of attrition bias. Comparing demographic variables revealed no statistical differences with regard to gender ratio, BMI, or household size. Trial completers, however, were found to be significantly younger (*P*=.02) than dropouts. Overall, our sensitivity analysis confirmed that missing data can be considered completely random in our trial and that no systematic error can thus be expected [70]. The large amount of missing data, however, reduced the statistical power of our analysis [69,70].

**Table 5:** Results of the sensitivity analysis.

Demographics and primary outcomes		Dropouts			Trial completers			*P* value^a^
		Value	n	Value		n		
**Gender, n (%)**			65			30	.66	
	Male	28 (43)		15 (50)				
	Female	37 (57)		15 (50)				
Age (years), median (IQR)		48 (20)	65	42 (18)		30	.02	
**BMI (kg/m^2^), n (%)**			65			30	.92^b^	
	>30	9 (14)		1 (3)				
	25-30	18 (28)		11 (37)				
	<25	38 (58)		18 (60)				
Household size, median (IQR)		2 (2)		3 (2)			.54	
**Physical activity, median (IQR)**								
	Walking (steps/day)	4897.83 (4286.13)	64	4077.50 (5499.05)		28	.95	
**Nutritional quality of food purchases, median (IQR)**								
	Total nutritional quality (FSA-NPS DI^c^ points)^d^	5.64 (3.87)	42	6.38 (4.26)		28	.24	

^a^Mann-Whitney *U* test.

^b^Mann-Whitney *U* test was performed comparing absolute BMI scores.

^c^FSA-NPS DI: British Food Standards Agency Nutrient Profiling System Dietary Index.

^d^FSA-NPS DI point scale: −15 most healthy to +40 least healthy.

### Qualitative Study Results

Among all participants who finished the trial, 15 participants were randomly selected to participate in semistructured video interviews. The selected participant demographics were similar to the overall trial demographics, and 47% (7/15) of the participants were female. The median age was 34 years (IQR 24), and the median household size was 2 (IQR 1). Overall, 53% (8/15) of the participants had received the FutureMe intervention and 47% (7/15) had received the control intervention.

### Ease of Use and Usefulness

While most participants experienced some challenges while signing up, particularly with connecting their loyalty cards to the app, they found using the app intuitive and easy. App functionalities were said to be simple and easily understandable.

The app is very simple, easy to read and intuitive to use. Really without a lot of bells and whistles or hidden features.

User #13, female

Overall, many users appreciated how easy the app was to use after the initial setup and that data synchronized automatically and effortlessly into the app.

Personally, I was motivated by the fact that the effort is relatively low, it is automatically synchronized, whether it is sports or shopping.

User #1, male

The app’s observed usefulness depended largely on how well participants perceived that tracking reflected their actual physical activity and food purchasing behavior. Participants who shopped mainly at the 2 participating grocery retailers and whose physical activity behavior mostly involved walking or running, rated app usefulness as very high. They felt that the app provided helpful insights, liked the color guidance on the overview tracking screen, and were inspired by the food tips.

Above all, to bring the bar up to green. As far as possible everywhere, but I haven’t achieved that yet.

User #8, male

I just found it very exciting with the purchases. […] I had the feeling that I buy very consciously and healthily, and sometimes I was in the orange and red area, where I thought just because I bought a pizza once, it drags the whole curve down. That is then nevertheless exciting and stimulates you to think about it and to consider whether it would not make sense to buy the wholewheat pizza dough or a vegan alternative instead of the normal pizza.

User #9, male

Participants who purchased their food at different grocery retailers or local markets, who regularly ate takeaways, or who shared the loyalty card with other family members found the app less useful. They felt that it did not reflect their actual behavior. They also stated that they would rate usefulness higher if tracking were more accurate.

It does not reflect things as they are. Because I don’t buy a lot of vegetables and fruit at these two retailers.

User #14, male

I was annoyed that my son just once again bought chips and peanuts.

User #5, female

### Participant-Avatar Relationship

Users who rated the app’s tracking accuracy high reported that the avatar motivated behavior change and that checking-in on the avatar was a key reason for logging into the app.

The avatar was super cool.

User #6, male

One app feature that motivated me was the avatar. I always clicked on the different items and checked to make sure that I improve on all categories.

User #4, female

Users liked that they could personalize their avatar and mentioned that the avatar somewhat resembled them. In contrast, most participants did not much like the avatar’s *mood* feature (ie, the facial expression depending on fruit, vegetable, and fiber consumption). In particular, when the avatar expressed sadness, users reported that this did not reflect how they felt. Also, more generally, users with less healthy food purchases or low physical activity behavior were more critical, with some users feeling discouraged by the avatar’s challenging feedback. They felt that some of the avatar’s reactions to their behaviors were exaggerated and did not reflect their state of health. This suggests that users struggled to fully understand that their avatar did not reflect their current health but rather their future health.

I could only partially identify with the avatar. I found especially that he gained weight very quickly. I couldn't understand why it was so fast. I had the feeling that he represented a worse image than my actual condition.

User #11, male

Users who rated the app’s tracking accuracy as low identified less with the avatar.

I mostly buy drinks and non-food items at the two participating retailers, so nothing that affects the avatar. Therefore, I can’t identify with it.

User #15, female

### Areas of Improvement

Users suggested improvements to the app’s tracking functionality; its analysis, insights, and feedback; and incentive schemes.

The most frequently mentioned area of improvement concerned broadening synchronized data sources and improving tracking accuracy. Although users appreciated that the automatic synchronization of food purchases required no personal effort, they wanted to be able to manually add data from other retailers, to include restaurant consumption, or to exclude foods purchased by other household members or unconsumed (food waste). Receipt scanning was mentioned multiple times as a preferred option for manually adding data, followed by drop-down functionalities. Further, users indicated that breaking down food purchases over a consumption period would improve app usefulness as certain items (eg, oil, salt, or condiments) are usually consumed over a longer period of time.

It would be cool if you could actually control the food analysis in the app via consumption and not the purchase, by dividing a product according to days or months in which I consume it.

User #1, male

Regarding activity tracking, users felt that including physical activity beyond the number of steps and improving app compatibility with leading fitness watches would be valuable improvements. Another related improvement often mentioned was improving data synchronization speed. In the FutureMe trial, food and physical activity data were updated daily. However, as an app login was required to synchronize loyalty card data with the app, it took approximately 2 days for food purchases to appear in the app. Users mentioned that they would have preferred to have food purchases available immediately after shopping.

Regarding the app’s analysis options, users liked the food tips, but felt that these were not updated regularly enough. Some users indicated that they would have liked to receive push notifications once new data were available in the app, or motivating push messages if their shopping or physical activity behavior deteriorated or improved. Furthermore, various participants mentioned that they would have liked to see the least healthy items in their shopping basket on a separate screen, as they felt that this would help to improve their shopping behavior, instead of merely receiving shopping tips for healthier alternatives.

You only see alternative product suggestions in the app. But it would be cool to see which products you purchased that you should consume less of. That would be more helpful.

User #4, female

Regarding feedback, some participants were discouraged by the avatar’s sometimes harsh language or appearance. They felt that empathetic positive language, even if they shopped unhealthily, would be more encouraging, which aligns with the motivational interviewing theory [74]. Furthermore, participants mentioned that animating the avatar or being able to adjust its clothing during the trial would improve user engagement with the overall app.

If you could dress the avatar however you want, I’d love that.

User #15, female

Lastly, some users suggested implementing an extrinsic incentive scheme in the app. Various users mentioned that an integrated financial bonus system or discounts for healthier food options would increase their motivation to use the app.

## Discussion

### Principal Findings

Insufficient physical activity and unhealthy diets are contributing to the rise in NCDs. Scalable preventative interventions are needed to combat this trend. To date, however, evidence of scalable mHealth interventions targeting both physical activity and nutritional improvements is scarce.

This is the first study to examine the impact of a future-self avatar mHealth intervention on physical activity and on the nutritional quality of food purchases. Using a 12-week RCT design, we found small statistically insignificant improvements in physical activity (median +242 steps/day) and small insignificant improvements in the nutritional quality of food purchases (median −1.28 FSA-NPS DI points). Low nutritional quality of food purchases is associated with a higher risk of developing chronic diseases and higher levels of obesity [60]. Moreover, increases in physical activity can lower the risk of metabolic syndromes and reduce all-cause mortality [75,76]. Our results provide some first evidence that scalable preventative mHealth interventions that use future-self avatars and automatic tracking of food choices via digital receipts from loyalty cards could contribute to reducing NCDs.

However, our study found no statistically significant changes in either of its primary outcomes over the course of the 12-week intervention. One underlying reason why we found no statistically significant improvements might be the reduced statistical power of our research due to the unexpectedly high attrition rate (n=95, 68.4%) and the resulting small sample at the end of the intervention. A meta-review of weight-loss interventions found that attrition rates vary significantly between studies (9%-90%) but found no consistent predictors [77]. Also, prior research has noted that high dropout rates are a “natural feature” of mHealth interventions [20]. Compared with traditional randomized controlled drug trials, adherence to an mHealth intervention is largely at the participant’s discretion [20]. This applies in particular when an intervention is not critical to participant well-being, as was the case in our preventative mHealth study. Further research with larger sample sizes is thus needed to understand whether a future-self avatar mHealth intervention can effectively reduce present bias and drive health behavior change.

To our knowledge, our study is the first mHealth intervention to implement fully automated food purchase tracking through loyalty cards in an RCT design outside of South Africa [25,26]. Based on a 12-week RCT and 15 semistructured interviews, we found that our technical solution is feasible and reliably tracks food purchases without requiring any participant effort after the initial setup of the FutureMe app. Prior work has found that individuals from northern European countries consume between 71.5% and 79.8% of their mean daily energy intake at home [78]. Analyzing food purchases through automized receipt tracking and a comprehensive nutritional product database is thus a promising method to guide improvements in overall nutrition and contributes to the need for automated and scalable nutrition tracking solutions [25,26]. Our study found that some participants were open to further improving the comprehensiveness of the analyzed food data by manually adding out-of-home food consumption and by increasing the number of included retailers. Future research should thus evaluate how to further optimize automated and semiautomated nutrition tracking systems so as to increase user retention.

Present bias offers a possible explanation as to why preventative mHealth interventions are less effective than curative mHealth interventions [15,32]. Our study thus examined whether a future-self avatar intervention can reduce present bias by increasing outcome expectancy. In our 12-week RCT, we found small statistically insignificant improvements in outcome expectancy in the FutureMe group, while this decreased in the control group. The results of our qualitative interviews show that some users did not understand that the FutureMe avatar reflected the future consequences of their current health behaviors. They assumed instead that the FutureMe avatar mirrors today’s health consequences. This effect could have undermined significant increases in outcome expectancy. However, we found that the future-self avatar intervention significantly increased intrinsic motivation (*P*=.03), while no increase occurred with the control intervention. Intrinsic motivation is associated with more sustainable behavior change than extrinsically motivated behavior [79]. Future-self avatars, as an element of gamification, might thus meaningfully contribute to improving the longer-term effectiveness of mHealth interventions, compared to more traditional text-based and numerical interfaces. Future research should explore how the interfaces and functionalities of future-self avatar mHealth interventions might be improved to more clearly communicate future health consequences, given the promising results this technology has produced in overcoming present bias in related research fields [31].

### Limitations

The strengths of our study are its use of objectively measured data for all primary and secondary outcomes, its rigorous research design, and its innovative future-self avatar interface and fully automated tracking system. However, it is not without limitations. First, the unexpectedly high attrition rate limited the sample size and statistical power of our study. Our qualitative study revealed that higher than expected dropout was partly caused by some users struggling to connect their grocery loyalty cards to the FutureMe app. Furthermore, users who shared the loyalty card with other members of their household or who did not shop at participating retailers for significant parts of their diets found the app less useful, potentially causing further dropout. We conducted a sensitivity analysis to test for attrition bias and found that participants who discontinued the app were not different from participants who finished the trial, based on their baseline demographics (with the exception of age), physical activity, and food purchasing behavior.

Second, the timing of our study may have impacted our results. Prior research has found that physical activity behavior is seasonally dependent [80]. Our study was conducted from November 2020 to April 2021, and the results thus may have been influenced by decreases in daylight and temperatures, as well as by unusual physical activity or food purchasing behavior due to the end-of-year holiday season and New Year’s resolutions. Also, the rise of the COVID-19 pandemic during the trial period may have impacted physical activity patterns and grocery purchases.

Third, we only tracked and analyzed food purchased at Switzerland’s 2 leading grocery retailers. The qualitative study demonstrated that this methodology does not comprehensively capture nutrition for certain user groups. However, the chosen retailers represent 69% of the grocery market in Switzerland [81]. While this share is considerable, noninclusion of other food purchases (eg, local farmers’ markets, butcher stores, or discounters) may have impacted our results. Also, data on purchased groceries automatically collected via loyalty cards usually only represent a subset of consumed products. In fact, the data set is missing information about food items sourced at other stores or markets, meals consumed in restaurants, and which of the purchased items were not consumed by the respective app user (eg, food waste or products eaten by household members other than the buyer). Furthermore, physical activity tracking was limited to measuring steps and did not include other forms of physical activity, which may have constrained our findings. Additionally, for iOS users, we only captured the number of steps recorded by their mobile phones. For Android users, we captured both the steps recorded by their mobile phones and those recorded by other devices connected to Google Fit.

Finally, the context of human diets is complex as individual dietary needs depend on many factors often unknown to an automatic system, including lifestyle, food allergies, and diet-related diseases, rendering static interventions ineffective. In the absence of a dietician or physician who could mitigate these challenges, automatic assessment interventions must be carefully designed, based on established nutritional guidelines (such as the Nutri-Score framework), and limited to healthy population samples.

### Conclusions

Our RCT found that a future-self avatar mHealth intervention significantly increased intrinsic motivation to lead a healthy lifestyle but did not lead to statistically significant improvements in physical activity levels and food purchasing behavior. Leveraging loyalty card data to track the nutritional quality of food purchases was found to be a feasible and accepted nutrition tracking technology and thus contributes to the search for scalable and automated tracking solutions. However, the high attrition rate and resulting small sample size of our study require further research to evaluate whether future-self avatar mHealth interventions can improve physical activity and food purchases at statistically significant levels. The use of future-self avatars may need to be modified in order to prevent attrition, reduce present bias, and promote sustainable behavior change.

## Appendix

Multimedia Appendix 1 Supplementary information on grocery loyalty card programs in Switzerland.

Multimedia Appendix 2 Calculation scheme for FutureMe avatar health states.

Multimedia Appendix 3 Scales for attitudinal and motivational constructs used in the FutureMe randomized controlled trial.

Multimedia Appendix 4 Primary outcomes by timepoint, within- and between-group comparisons.

Multimedia Appendix 5 Secondary outcomes by timepoint and group, within- and between-group comparisons.

Multimedia Appendix 6 CONSORT-eHEALTH checklist (V 1.6.2).

## Abbreviations

FSA-NPS DI: British Food Standards Agency Nutrient Profiling System Dietary Index
HAPA: Health Action Process Approach
mHealth: mobile health
NCD: noncommunicable disease
RCT: randomized controlled trial
WHO: World Health Organization
