# Supplementary material for: The Effect of a Future-Self Avatar Mobile Health Intervention (FutureMe) on Physical Activity and Food Purchases: Randomized Controlled Trial
Source: J Med Internet Res. 2022 Jul 7;24(7):e32487. doi: 10.2196/32487 (PMC9305430; doi:10.2196/32487)
Supplement: Multimedia Appendix 1 [file jmir_v24i7e32487_app1.pdf]

Appendix 1: Supplementary information on grocery loyalty card programs in Switzerland.

Reach: Grocery loyalty cards are widely used in Switzerland. Coop Supercard is used by over 4 million users [1] which is in line with the number of private households in Switzerland [2]. Migros's Cumulus card is reported to be used in 80% of all grocery purchases performed at Migros [3].

Loyalty data used in this study: For this study, we used the timepoint of each grocery purchase, the nutritional information of each grocery item purchased declared in line with EU-standards for nutritional labelling (EU1169/2011), and the weight of each grocery item, measured in grams.

References

1. Luzerner Zeitung. Supercard-Punkte belasten Coop-Bilanz, Migros macht es mit Cumulus besser. 2015 URL: https://www.luzernerzeitung.ch/wirtschaft/supercard-punkte-belasten-coop-bilanz-migros-macht-es-mit-cumulus-besser-ld.1676001 [accessed 2021-10-18]

2. Federal Statistical Office. Private households by household type. 2021. URL: https://www.bfs.admin.ch/bfs/en/home/statistics/population/effectif-change.assetdetail.16005651.html [accessed 2021-10-18]

3. Migros. Cumulus: Die beliebteste Kundenkarte der Schweiz feiert ihren 20. Geburtstag. 2017. URL: https://www.migros.ch/de/unternehmen/medien/mitteilungen/show/news/medienmitteilungen/2017/cumulus-20-geburtstag.html [accessed 2021-10-18]
