# Supplementary material for: The Effect of a Future-Self Avatar Mobile Health Intervention (FutureMe) on Physical Activity and Food Purchases: Randomized Controlled Trial
Source: J Med Internet Res. 2022 Jul 7;24(7):e32487. doi: 10.2196/32487 (PMC9305430; doi:10.2196/32487)
Supplement: Multimedia Appendix 2 [file jmir_v24i7e32487_app2.pdf]

Appendix 2: Calculation scheme for FutureMe avatar health states.

	State 1<br>Very healthy	State 2<br>Healthy	State 3<br>Average
Physical Activity in Steps/Day [45]	>12,500	10,000 - 12,499	9,999 - 7,499
Salt in FSA-NPS-DI [60]	0-2	2-4	4-6
Fruit, Vegetables, legumes & nuts in FSA-NPS-DI [60]	4-5	3-4	2-3
Dietary Fiber in FSA-NPS-DI [60]	4-5	3-4	2-3
Saturated fatty acids [illegible]	0-2	2-4	4-6
