# Supplementary material for: The Effect of a Future-Self Avatar Mobile Health Intervention (FutureMe) on Physical Activity and Food Purchases: Randomized Controlled Trial
Source: J Med Internet Res. 2022 Jul 7;24(7):e32487. doi: 10.2196/32487 (PMC9305430; doi:10.2196/32487)
Supplement: Multimedia Appendix 3 [file jmir_v24i7e32487_app3.pdf]

Appendix 3: Scales for attitudinal and motivational constructs used in the FutureMe randomized-controlled trial.

Legend: 1=completely disagree, 7=completely agree

Motivational self-efficacy (adapted from Schwarzer et al. 2007) [61]

**A**	**In my everyday life, …**	1	2	3	4	5	6	7
A1	… I manage to exercise three times a week for 30 min.							
A2	… I can make sure that I exercise at least once a week							
A3	… I know how to shop healthily							
A4	… I make sure I have a balanced shopping cart when I shop.							

Recovery self-efficacy (adapted from Schwarzer et al. 2007) [61]

**B**	**Imagine you come back from vacation and need to get back into your daily routine.**<br>**After my vacation, I'm sure that…**	1	2	3	4	5	6	7
B1	… I'll go back to balanced shopping, even if I have to get used to it again.							
B2	… I'll get back to regular exercise, even if I don't see immediate results.							

Outcome expectancy (adapted from Renner and Schwarzer, 2005) [62]

**C**	**Do you think your behavior today can affect your health as you age?**<br>**I believe that…**	1	2	3	4	5	6	7
C1	… I can positively influence my health in old age with my current exercise and shopping behavior.							
C2	… I am reducing my risk of obesity in old age with my current exercise and shopping behavior.							
C3	… I am reducing my risk of developing diabetes in old age with my current exercise and shopping behavior.							
C4	… I am lowering my risk of suffering from cardiovascular disease in old age with my current exercise and shopping behavior.							
C5	… I can improve my overall mental well-being in old age with my current exercise and shopping behavior.							
C6	… I can improve my mobility in old age with my current exercise and shopping behavior.							

Intrinsic & extrinsic motivation (adapted from Williams, Ryan and Deci, 2016) [63]

**D**	**Why is it important for you to exercise and shop healthily?**<br><br>**I exercise and shop healthily because…**	1	2	3	4	5	6	7
D1	… I personally believe it's best for my health.							
D2	… it's consistent with my life goals.							
D3	… it is very important to be as healthy as possible.							
D4	… I want to see positive metrics on my activity tracker and health app.							
D5	… I want others to see that I am being healthy.							
D6	… I want praise from others.							
