# Supplementary material for: The Effect of a Future-Self Avatar Mobile Health Intervention (FutureMe) on Physical Activity and Food Purchases: Randomized Controlled Trial
Source: J Med Internet Res. 2022 Jul 7;24(7):e32487. doi: 10.2196/32487 (PMC9305430; doi:10.2196/32487)
Supplement: Multimedia Appendix 4 [file jmir_v24i7e32487_app4.pdf]

Appendix 4: Primary outcomes by timepoint, within- and between-group comparisons.

Outcome variables	Within-group comparison vs. baseline						Between-group comparison							
	Baseline vs. T1		Baseline vs. T2		Baseline vs. EoS		Baseline[b]		T1[c]		T2[d]		EoS[e]	
	Z	*P* value	Z	*P* value	Z	*P* value	U	*P* value	U	*P* value	U	*P* value	U	*P* value
**Physical Activity, median (IQR)**														
Walking (in steps/day)							965	.53	656	.34	212	.93	105	.92
Avatar	-0.3	.79	-0.283	.80	-0.39	.73								
Control	-0.9	.38	-0.438	.68	-0.57	.60								
**Nutritional quality of food purchases, median (IQR)**														
Total nutritional quality (in FSA-NPS-DI-points)[a]							470	.14	505	.29	660	.44	796	.02
Avatar	-1.8	.07	-0.299	.77	-0.56	.58								
Control	-0.4	.70	-0.213	.83	-1.26	.21								

[a] FSA-NPS-DI point scale: -15 most healthy to +40 least healthy.

[b] Baseline: For steps baseline defined as average steps/day 6 days prior to enrolling in the trial;

for shopping-related outcome variables baseline is defined as nutritional value of all foods purchased within the 4 weeks before the trial.

[c] T1: Week 1-4 average values

[d] T2: Week 5-8 average values

[e] EoS: End of Study, defined as mean value week 9-12.

Comment: *P* value reported as exact significances due to small sample sizes.
