# Supplementary material for: The Effect of a Future-Self Avatar Mobile Health Intervention (FutureMe) on Physical Activity and Food Purchases: Randomized Controlled Trial
Source: J Med Internet Res. 2022 Jul 7;24(7):e32487. doi: 10.2196/32487 (PMC9305430; doi:10.2196/32487)
Supplement: Multimedia Appendix 5 [file jmir_v24i7e32487_app5.pdf]

# **Appendix 5:** Secondary outcomes by timepoint and group, within- and between-group comparisons.

| Outcome variables | 4-week period actuals | | | | | | | | Within-group comparison vs. baseline[h] | | | | | | Between-group comparison[i] | | | | | | | |
|---|---|---|---|---|---|---|---|---|---|---|---|---|---|---|---|---|---|---|---|---|---|---|
| | | | | | | | | | Baseline vs. T1 | | Baseline vs. T2 | | Baseline vs. EoS | | Baseline | | T1 | | T2 | | EoS | |
| | Baseline[d] | n | T1[e] | n | T2[f] | n | EoS[g] | n | Z | *P* value | Z | *P* value | Z | *P* value | U | *P* value | U | *P* value | U | *P* value | U | *P* value |
| **Nutritional sub-categories, median (IQR)** | | | | | | | | | | | | | | | | | | | | | | |
| Sugars (in FSA-NPS-DI points)[a] | | | | | | | | | | | | | | | 565 | .73 | 484 | .19 | 592.5 | .97 | 823.5 | .006 |
| Avatar | 0.83 (1.08) | 27 | 0.95 (0.90) | 20 | 1.06 (0.39) | 14 | 0.89 (0.47) | 13 | -0.561 | .58 | -1.542 | .12 | -0.56 | .58 | | | | | | | | |
| Control | 0.91 (1.31) | 40 | 0.65 (0.86) | 34 | 0.74 (1.07) | 19 | 0.55 (1.07) | 14 | -0.994 | .32 | -0.471 | .64 | -0.3 | .76 | | | | | | | | |
| Saturated fatty acids (in FSA-NPS-DI points)[a] | | | | | | | | | | | | | | | 412 | .03 | 493.5 | .23 | 542 | .54 | 824.5 | .006 |
| Avatar | 3.82 (2.83) | 27 | 3.44 (1.34) | 20 | 3.53 (0.78) | 14 | 3.47 (1.36) | 13 | -1.478 | .14 | 0.00 | 1.00 | -0.13 | .90 | | | | | | | | |
| Control | 2.93 (1.37) | 40 | 3.06 (2.30) | 34 | 2.07 (2.23) | 19 | 3.10 (1.66) | 14 | -0.128 | .90 | -0.731 | .47 | -1.45 | .15 | | | | | | | | |
| Sodium (in FSA-NPS-DI points)[a] | | | | | | | | | | | | | | | 527 | .42 | 458 | .11 | 614 | .82 | 732 | .10 |
| Avatar | 2.77 (1.56) | 27 | 2.76 (1.82) | 20 | 2.43 (1.50) | 14 | 2.21 (1.46) | 13 | -0.818 | .41 | -0.128 | .90 | -0.21 | .83 | | | | | | | | |
| Control | 2.42 (1.37) | 40 | 2.40 (1.69) | 34 | 2.31 (1.18) | 19 | 2.86 (2.13) | 14 | 0.00 | 1.00 | -0.471 | .64 | -0.13 | .90 | | | | | | | | |
| Fruit, vegetables, legumes and nuts (in FSA-NPS-DI points)[b] | | | | | | | | | | | | | | | 555 | .64 | 449 | .08 | 722 | .13 | 681 | .31 |
| Avatar | 0.28 (0.53) | 27 | 0.22 (0.32) | 20 | 0.15 (0.49) | 14 | 0.20 (0.28) | 13 | -0.561 | .58 | -0.561 | .58 | -0.56 | .58 | | | | | | | | |
| Control | 0.31 (0.47) | 40 | 0.12 (0.22) | 34 | 0.20 (0.52) | 19 | 0.10 (0.17) | 14 | -1.274 | .20 | -0.77 | .44 | -1.36 | .17 | | | | | | | | |
| Fibers (in FSA-NPS-DI points)[b] | | | | | | | | | | | | | | | 611 | .85 | 558.5 | .67 | 722 | .13 | 709 | .17 |
| Avatar | 1.49 (0.70) | 27 | 1.44 (0.95) | 20 | 1.60 (0.54) | 14 | 1.69 (0.98) | 13 | -1.172 | .24 | -0.471 | .64 | -0.64 | .52 | | | | | | | | |
| Control | 1.56 (0.83) | 40 | 1.84 (1.17) | 34 | 1.91 (1.05) | 19 | 1.75 (0.94) | 14 | -0.644 | .52 | 1.733 | .08 | -0.13 | .90 | | | | | | | | |
| Protein (in FSA-NPS-DI points)[b] | | | | | | | | | | | | | | | 509.5 | .31 | 579.5 | .86 | 543 | .54 | 711 | .17 |
| Avatar | 3.20 (0.70) | 27 | 2.84 (1.18) | 20 | 3.28 (0.62) | 14 | 3.26 (0.60) | 13 | -0.459 | .65 | -0.644 | .52 | -0.39 | .70 | | | | | | | | |
| Control | 3.15 (1.03) | 40 | 3.15 (0.91) | 34 | 2.88 (0.92) | 19 | 3.42 (1.17) | 14 | -0.731 | .47 | -1.174 | .24 | -0.82 | .41 | | | | | | | | |
| **User engagement, median (IQR)** | | | | | | | | | | | | | | | | | | | | | | |
| Logins (logins/week) | | | | | | | | | | | | | | | - | - | 729.00 | .28 | 132.50 | .93 | 50.00 | .47 |
| Avatar | - | - | 5.00 (10.00) | 36 | 1.00 (3.00) | 15 | 1.00 (1.00) | 11 | - | - | - | - | - | - | | | | | | | | |
| Control | - | - | 4.00 (10.00) | 47 | 1.00 (3.00) | 18 | 2.00 (2.00) | 11 | - | - | - | - | - | - | | | | | | | | |
| **Attitudes & motivation, median (IQR)** | | | | | | | | | | | | | | | | | | | | | | |
| Motivational self-efficacy[c] | | | | | | | | | | | | | | | 1080.00 | .81 | - | - | - | - | 47.00 | .65 |
| Avatar | 6.00 (1.31) | 42 | - | - | - | - | 6.00 (1.38) | 9 | - | - | - | - | 0.000 | 1.00 | - | - | - | - | - | - | - | - |
| Control | 6.00 (1.50) | 53 | - | - | - | - | 6.13 (1.00) | 12 | - | - | - | - | -0.240 | .83 | - | - | - | - | - | - | - | - |
| Recovery self-efficacy[c] | | | | | | | | | | | | | | | 1061.00 | .69 | - | - | - | - | 24.00 | .03 |
| Avatar | 6.00 (1.50) | 42 | - | - | - | - | 7.00 (1.00) | 9 | - | - | - | - | -0.431 | .67 | - | - | - | - | - | - | - | - |
| Control | 6.00 (1.50) | 53 | - | - | - | - | 5.75 (1.25) | 12 | - | - | - | - | -1.725 | .13 | - | - | - | - | - | - | - | - |
| Outcome expectancy[c] | | | | | | | | | | | | | | | 1110.50 | .99 | - | - | - | - | 39.00 | .31 |
| Avatar | 6.10 (1.65) | 42 | - | - | - | - | 7.00 (2.08) | 9 | - | - | - | - | -0.338 | .81 | - | - | - | - | - | - | - | - |
| Control | 6.20 (1.60) | 53 | - | - | - | - | 5.67 (1.62) | 12 | - | - | - | - | -0.196 | .87 | - | - | - | - | - | - | - | - |
| Intrinsic motivation[c] | | | | | | | | | | | | | | | 1073.50 | .77 | - | - | - | - | 32.00 | .13 |
| Avatar | 5.83 (1.75) | 42 | - | - | - | - | 6.00 (1.67) | 9 | - | - | - | - | -2.217 | .03 | - | - | - | - | - | - | - | - |
| Control | 6.00 (1.50) | 53 | - | - | - | - | 5.50 (1.50) | 12 | - | - | - | - | -1.491 | .16 | - | - | - | - | - | - | - | - |
| Extrinsic motivation[c] | | | | | | | | | | | | | | | 871.00 | .07 | - | - | - | - | 47.50 | .65 |
| Avatar | 2.67 (2.00) | 42 | - | - | - | - | 3.67 (2.50) | 9 | - | - | - | - | -1.612 | .12 | - | - | - | - | - | - | - | - |
| Control | 3.33 (2.00) | 53 | - | - | - | - | 4.00 (2.08) | 12 | - | - | - | - | -0.060 | 1.00 | - | - | - | - | - | - | - | - |

[a] FSA-NPS-DI point scale: 0 most healthy to +10 least healthy.
[b] FSA-NPS-DI point scale: 0 least healthy to +5 most healthy.
[c] Likert scale: 1 completely disagree to 7 coompletely agree.
[d]Baseline: for steps defined as average steps/day 6 weeks prior to enrolling in the trial; for shopping-related outcome variables baseline is defined as nutritional value of all foods purchased within the 4 weeks before the trial.
[e]T1: Week 1-4 average values.
[f]T2: Week 5-8 average values.
[g]EoS: End of Study, defined as mean value week 9-12.
[h]Wilcoxon sign-rank test was performed to detect statistical differences.
[i]Mann-Whitney U test was performed to detect statistical differences.
