# Supplementary material for: The Effect of a Future-Self Avatar Mobile Health Intervention (FutureMe) on Physical Activity and Food Purchases: Randomized Controlled Trial
Source: J Med Internet Res. 2022 Jul 7;24(7):e32487. doi: 10.2196/32487 (PMC9305430; doi:10.2196/32487)
Supplement: Multimedia Appendix 6 [file jmir_v24i7e32487_app6.pdf]

CONSORT-EHEALTH Checklist V1.6.2 Report	Manuscript Number	32487
**(based on CONSORT-EHEALTH V1.6), available at [http://tinyurl.com/consort-ehealth-v1-6].**		
**Date completed**		
8/10/2021 3:53:52		
**by**		
Annette Mönninghoff		
The Effect of a Future-Self Avatar mHealth Intervention on Physical Activity and Food Purchases: The FutureMe Randomized Controlled Trial		
TITLE		
**1a-i) Identify the mode of delivery in the title**		
Yes, the study is mobile-based. See the part in the title 'a Future-Self Avatar mHealth Intervention'.		
**1a-ii) Non-web-based components or important co-interventions in title**		
**1a-iii) Primary condition or target group in the title**		
Yes, we mentioned the primary conditions/objective 'physical activity and food purchases'.		
ABSTRACT		
**1b-i) Key features/functionalities/components of the intervention and comparator in the METHODS section of the ABSTRACT**		
Yes, 'The intervention group received the FutureMe intervention—a physical activity and food purchase tracking mobile phone application that uses a future-self avatar as the primary interface and provides participants with personalized food basket analysis and shopping tips. The control group received a conventional, text- and graphic-based primary interface intervention. '		
**1b-ii) Level of human involvement in the METHODS section of the ABSTRACT**		
Yes, 'We pioneered a novel system to track nutrition leveraging digital receipts from loyalty card data analyzing food purchases in a fully automated way'.		
**1b-iii) Open vs. closed, web-based (self-assessment) vs. face-to-face assessments in the METHODS section of the ABSTRACT**		
Yes, we cover it in the manuscript as there is a word limit of the abstract.		
**1b-iv) RESULTS section in abstract must contain use data**		
Yes, 'We recruited 167 participants; 95 eligible participants were randomized into either the intervention (n=42) or control group (n=53). The median age was 44.00 years (IQR 19.00), and the gender ratio was balanced (female 52/95, 55%). Attrition was unexpectedly high with only 30 participants completing the intervention, negatively impacting the statistical power of our study.'		
**1b-v) CONCLUSIONS/DISCUSSION in abstract for negative trials**		
Yes, we cover it in detail in the manuscript as there is a word limit of the abstract.		
INTRODUCTION		
**2a-i) Problem and the type of system/solution**		
Yes, see the section 'Non-Communicable Diseases and Risk Factors'.		
**2a-ii) Scientific background, rationale: What is known about the (type of) system**		
Yes, see the subsections from 'Physical Activity and Salt Reduction mHealth Interventions' until 'Future-Self Avatars to Overcome Present Bias and Increase Risk Awareness'.		
**Does your paper address CONSORT subitem 2b?**		
Yes, see the subsection 'Objectives'.		
METHODS		
**3a) CONSORT: Description of trial design (such as parallel, factorial) including allocation ratio**		
Yes, see the subsection 'Study Design'.		

**3b) CONSORT: Important changes to methods after trial commencement (such as eligibility criteria), with reasons**		
No adjustments made after trial commencement.		
**3b-i) Bug fixes, Downtimes, Content Changes**		
**4a) CONSORT: Eligibility criteria for participants**		
Yes, see the subsection 'Study Population'.		
**4a-i) Computer / Internet literacy**		
**4a-ii) Open vs. closed, web-based vs. face-to-face assessments:**		
Yes, see the subsection 'Study Design'.		
**4a-iii) Information giving during recruitment**		
**4b) CONSORT: Settings and locations where the data were collected**		
Yes, see the subsection 'Study Design'.		
**4b-i) Report if outcomes were (self-)assessed through online questionnaires**		
Yes, see the subsection 'Study Design'.		
**4b-ii) Report how institutional affiliations are displayed**		
**5) CONSORT: Describe the interventions for each group with sufficient details to allow replication, including how and when they were actually administered**		
**5-i) Mention names, credential, affiliations of the developers, sponsors, and owners**		
**5-ii) Describe the history/development process**		
**5-iii) Revisions and updating**		
**5-iv) Quality assurance methods**		
**5-v) Ensure replicability by publishing the source code, and/or providing screenshots/screen-capture video, and/or providing flowcharts of the algorithms used**		
**5-vi) Digital preservation**		
**5-vii) Access**		
Yes, see the subsections 'FutureMe Intervention' and 'Control Intervention'.		
**5-viii) Mode of delivery, features/functionalities/components of the intervention and comparator, and the theoretical framework**		
Yes, see the subsections 'FutureMe Intervention' and 'Control Intervention'.		
**5-ix) Describe use parameters**		
**5-x) Clarify the level of human involvement**		
**5-xi) Report any prompts/reminders used**		

No prompts/reminders were used, as described in section “FutureMe Intervention” and “Control Intervention".		
**5-xii) Describe any co-interventions (incl. training/support)**		
No co-interventions were performed. As described in section “FutureMe Intervention” and “Control Intervention", the intervention solely consisted of the smartphone application.		
**6a) CONSORT: Completely defined pre-specified primary and secondary outcome measures, including how and when they were assessed**		
Yes, see the subsection 'Outcomes and Measurement'.		
**6a-i) Online questionnaires: describe if they were validated for online use and apply CHERRIES items to describe how the questionnaires were designed/deployed**		
**6a-ii) Describe whether and how “use” (including intensity of use/dosage) was defined/measured/monitored**		
**6a-iii) Describe whether, how, and when qualitative feedback from participants was obtained**		
**6b) CONSORT: Any changes to trial outcomes after the trial commenced, with reasons**		
Yes, see the subsection 'Study Design'.		
**7a) CONSORT: How sample size was determined**		
**7a-i) Describe whether and how expected attrition was taken into account when calculating the sample size**		
**7b) CONSORT: When applicable, explanation of any interim analyses and stopping guidelines**		
Yes, see the subsection 'Outcomes and Measurement'.		
**8a) CONSORT: Method used to generate the random allocation sequence**		
See section study design “After downloading the mobile app, answering a survey on eligibility criteria, and consenting to the data privacy statement, participants were randomized into an intervention or a control group (1:1 ratio) via a random allocation algorithm programmed into the app".		
**8b) CONSORT: Type of randomisation; details of any restriction (such as blocking and block size)**		
See section study design “After downloading the mobile app, answering a survey on eligibility criteria, and consenting to the data privacy statement, participants were randomized into an intervention or a control group (1:1 ratio) via a random allocation algorithm programmed into the app."		
**9) CONSORT: Mechanism used to implement the random allocation sequence (such as sequentially numbered containers), describing any steps taken to conceal the sequence until interventions were assigned**		
See section study design “After downloading the mobile app, answering a survey on eligibility criteria, and consenting to the data privacy statement, participants were randomized into an intervention or a control group (1:1 ratio) via a random allocation algorithm programmed into the app."'		
**10) CONSORT: Who generated the random allocation sequence, who enrolled participants, and who assigned participants to interventions**		
See section study design “After downloading the mobile app, answering a survey on eligibility criteria, and consenting to the data privacy statement, participants were randomized into an intervention or a control group (1:1 ratio) via a random allocation algorithm programmed into the app." The algorithm was programmed by an independent programmer who developed the front-end app design. No researcher had any influence on group allocation. Allocation was concealed as it was fully automated and there was no human contact with any trial participants throughout the trial.		
**11a) CONSORT: Blinding - If done, who was blinded after assignment to interventions (for example, participants, care providers, those assessing outcomes) and how**		
**11a-i) Specify who was blinded, and who wasn’t**		
See the subsection 'Study Design'. While the data is anonymous, the researchers were blinded. Participants knew which group they belong to because the user interfaces of different groups are different.		
**11a-ii) Discuss e.g., whether participants knew which intervention was the “intervention of interest” and which one was the “comparator”**		
**11b) CONSORT: If relevant, description of the similarity of interventions**		
Yes, see the subsection 'Study Design'.		

**12a) CONSORT: Statistical methods used to compare groups for primary and secondary outcomes**		
Yes, see the subsection 'Statistical Analysis'.		
**12a-i) Imputation techniques to deal with attrition / missing values**		
Yes, see the subsection 'Statistical Analysis'.		
**12b) CONSORT: Methods for additional analyses, such as subgroup analyses and adjusted analyses**		
Yes, see the subsection 'Statistical Analysis'.		
RESULTS		
**13a) CONSORT: For each group, the numbers of participants who were randomly assigned, received intended treatment, and were analysed for the primary outcome**		
Yes, see the subsection 'Demographic Data and Baseline Characteristics'.		
**13b) CONSORT: For each group, losses and exclusions after randomisation, together with reasons**		
Yes, see the subsection 'Demographic Data and Baseline Characteristics'.Figure 4: Consort flow chart.		
**13b-i) Attrition diagram**		
**14a) CONSORT: Dates defining the periods of recruitment and follow-up**		
Yes, see the subsection 'Demographic Data and Baseline Characteristics'.Figure 4: Consort flow chart.		
**14a-i) Indicate if critical “secular events” fell into the study period**		
**14b) CONSORT: Why the trial ended or was stopped (early)**		
Yes, see the subsection 'Demographic Data and Baseline Characteristics'.		
**15) CONSORT: A table showing baseline demographic and clinical characteristics for each group**		
Yes, see the subsection 'Demographic Data and Baseline Characteristics'.		
**15-i) Report demographics associated with digital divide issues**		
Yes, see the subsection 'Demographic Data and Baseline Characteristics'.		
**16a) CONSORT: For each group, number of participants (denominator) included in each analysis and whether the analysis was by original assigned groups**		
**16-i) Report multiple “denominators” and provide definitions**		
Yes, see the subsection 'Demographic Data and Baseline Characteristics'.		
**16-ii) Primary analysis should be intent-to-treat**		
**17a) CONSORT: For each primary and secondary outcome, results for each group, and the estimated effect size and its precision (such as 95% confidence interval)**		
Yes, see the subsection 'Demographic Data and Baseline Characteristics'.		
**17a-i) Presentation of process outcomes such as metrics of use and intensity of use**		
**17b) CONSORT: For binary outcomes, presentation of both absolute and relative effect sizes is recommended**		
Yes, see the subsection 'Results'.		
**18) CONSORT: Results of any other analyses performed, including subgroup analyses and adjusted analyses, distinguishing pre-specified from exploratory**		
Yes, see the subsection 'Results'.		
**18-i) Subgroup analysis of comparing only users**		
**19) CONSORT: All important harms or unintended effects in each group**		

Yes, see the subsection 'Results'.		
**19-i) Include privacy breaches, technical problems**		
**19-ii) Include qualitative feedback from participants or observations from staff/researchers**		
DISCUSSION		
**20) CONSORT: Trial limitations, addressing sources of potential bias, imprecision, multiplicity of analyses**		
**20-i) Typical limitations in ehealth trials**		
Yes, see the subsection 'Limitations'.		
**21) CONSORT: Generalisability (external validity, applicability) of the trial findings**		
**21-i) Generalizability to other populations**		
**21-ii) Discuss if there were elements in the RCT that would be different in a routine application setting**		
**22) CONSORT: Interpretation consistent with results, balancing benefits and harms, and considering other relevant evidence**		
**22-i) Restate study questions and summarize the answers suggested by the data, starting with primary outcomes and process outcomes (use)**		
Yes, see the subsection 'Principal Findings'.		
**22-ii) Highlight unanswered new questions, suggest future research**		
Other information		
**23) CONSORT: Registration number and name of trial registry**		
Yes, see the subsection 'Study Design'. "The trial has been registered on ClinicalTrials.gov (NCT04505124)."		
**24) CONSORT: Where the full trial protocol can be accessed, if available**		
Yes, see the subsection 'Study Design'.		
**25) CONSORT: Sources of funding and other support (such as supply of drugs), role of funders**		
Yes, see the subsection 'Conflicts of Interest'.		
**X26-i) Comment on ethics committee approval**		
**x26-ii) Outline informed consent procedures**		
**X26-iii) Safety and security procedures**		
**X27-i) State the relation of the study team towards the system being evaluated**		
